# Expression and clinical prognostic value of m6A RNA methylation modification in breast cancer

**DOI:** 10.1186/s40364-021-00285-w

**Published:** 2021-04-29

**Authors:** Fangchao Zheng, Feng Du, Haili Qian, Jiuda Zhao, Xue Wang, Jian Yue, Nanlin Hu, Yiran Si, Binghe Xu, Peng Yuan

**Affiliations:** 1 Department of Medical Oncology, National Cancer Center/National Clinical Research Center for Cancer/Cancer Hospital, Chinese Academy of Medical Sciences and Peking Union Medical College, No. 17 Panjiayuan Nanli, Beijing, 100021 China; 2 Key Laboratory of Carcinogenesis and Translational Research (Ministry of Education/Beijing), The VIPII Gastrointestinal Cancer Division of Medical Department, Peking University Cancer Hospital and Institute, Beijing, 100021 China; 3 State Key Laboratory of Molecular Oncology, Cancer Hospital/Institute, Chinese Academy of Medical Sciences and Peking Union Medical College, Beijing, 100021 China; 4 Breast Disease Diagnosis and Treatment Center, Affiliated Hospital of Qinghai University & Affiliated Cancer Hospital of Qinghai University, Xining, 810000 China; 5 Department of VIP Medical Services, National Cancer Center/National Clinical Research Center for Cancer/Cancer Hospital, Chinese Academy of Medical Sciences and Peking Union Medical College, Beijing, 100021 China

**Keywords:** N6-methyladenosine modification, Methylation, Breast cancer, CBLL1, Prognosis

## Abstract

**Background:**

N6-methyladenosine(m6A) methylation modification affects the tumorigenesis, progression, and metastasis of breast cancer (BC). However, the expression characteristics and prognostic value of m6A modification in BC are still unclear. We aimed to evaluate the relationship between m6A modification and clinicopathological characteristics, and to explore the underlying mechanisms.

**Methods:**

Three public cohorts and our clinical cohort were included: 1091 BC samples and 113 normal samples from the TCGA database, 1985 BC samples from the METABRIC database, 1764 BC samples from the KM Plotter website, and 134 BC samples of our clinical cohort. We collected date from these cohorts and analyzed the genetic expression, gene-gene interactions, gene mutations, copy number variations (CNVs), and clinicopathological and prognostic features of 28 m6A RNA regulators in BC.

**Results:**

This study demonstrated that some m6A regulators were significantly differenially expressed in BCs and their adjacent tissues, and also different in various molecular types. All 28 studied m6A regulators exhibited interactions. KIAA1429 had the highest mutation frequency. CNVs of m6A regulators were observed in BC patients. The expression of the m6A regulators was differentially associated with survival of BC. Higher CBLL1 expression was associated with a better prognosis in BC than lower CBLL1 expression. Functional analysis showed that CBLL1 was related to the ESR1-related pathway, apoptosis-related pathway, cell cycle pathway and immune-related pathway in BC.

**Conclusions:**

m6A RNA modification modulated gene expression and thereby affected clinicopathological features and survival outcomes in BC. CBLL1 may be a promising prognostic biomarker for BC patients.

**Supplementary Information:**

The online version contains supplementary material available at 10.1186/s40364-021-00285-w.

## Highlights

1. m6A RNA methylation is associated with the differential gene expression, CNV, molecular typing, and clinicopathological and prognostic features of breast cancer.
2. CBLL1 is correlated with better prognosis in breast cancer.
3. This is the first study to report m6A RNA expression characteristics and prognostic value in breast cancers based on three public cohorts and our clinical cohort.

## Introduction

Breast carcinoma (BC) is the most commonly diagnosed malignant tumor and the leading cause of cancer-related deaths annually in women annually; thus, it is a serious threat to the health of females [1]. The outcome for BC patients worldwide have improved because of the development of medical therapy, surgical therapy, and novel therapeutic interventions and the use of newer molecular typing techniques. Despite these efforts, BC continues to have a 15% of cancer-related death rate following treatment according to GLOBOCAN2018, and the mortality is still not optimistic [1]. In addition, WHO Cancer Tomorrow predicted that approximately 817, 361 females will die from BC by 2030. That is, the prognosis of BC still remains dismal. Thus, there is an urgent need exists to develop new treatment options to improve survival for BC patients, particularly those with disease or recurrent metastasis.

RNA modification, especially N6-methyladenosine (m6A) modification, have provided a more effective method and a new prospects in the treatment of BC [2]. The m6A modification is a highly abundant and conservative messenger RNA modification in mammals that consists of three vital components, as follows: writers, which are also termed methyltransferases; erasers, which are demethylases that remove m6A modifications; and readers, which recognize m6A-modified sites and regulate m6A modifications [2]. In general, m6A RNA modifications regulate RNA termination codons, 5’cap structure, and the 3′ untranslated region (UTR), positively or negatively affecting tumorigenesis, tumor differentiation, tumor proliferation, tumor invasion and tumor metastasis of BC [2, 3].

Growing evidence has demonstrated that m6A modification is closely associated with tumorigenesis, tumor differentiation, tumor proliferation, tumor invasion and worse survival in BC patients, including METTL3, METTL14, WTAP, ALKBH5, IGF2BP2, IGF2BP3, and FTO [3–14]. In addition, the m6A regulator IGF2BP3 was associated with reduced cell apoptosis, larger tumor sizes, higher grade, higher clinical stage, necrosis, and CK5/6 expression and further worsened the DFS and OS of BC patients [4, 15, 16]. However, the m6A regulator EIF3A had no association with age, tumor size, or differentiation grade. Unfortunately, data published on m6A modification in BC are partly conflicting and highly heterogeneous. Most studies have investigated partial mechanisms and capabilities, and the gene is relatively simplistic. Some studies evaluated the prognostic value of m6A regulators in BC patients, but significantly fewer tissue samples were included.

In the present study, we clarified the biological mechanism of all 28 m6A regulators and evaluated the associations with the clinicopathological features and prognosis in BC patients based on The Cancer Genome Atlas (TCGA), Molecular Taxonomy of Breast Cancer International Consortium (METABRIC), KM Plotter website, and one clinical cohort. The aim of this study is to identify potential new therapeutic targets and improve the prognosis of BC patients.

## Materials and methods

### Data acquisition

**Fig. 1:**
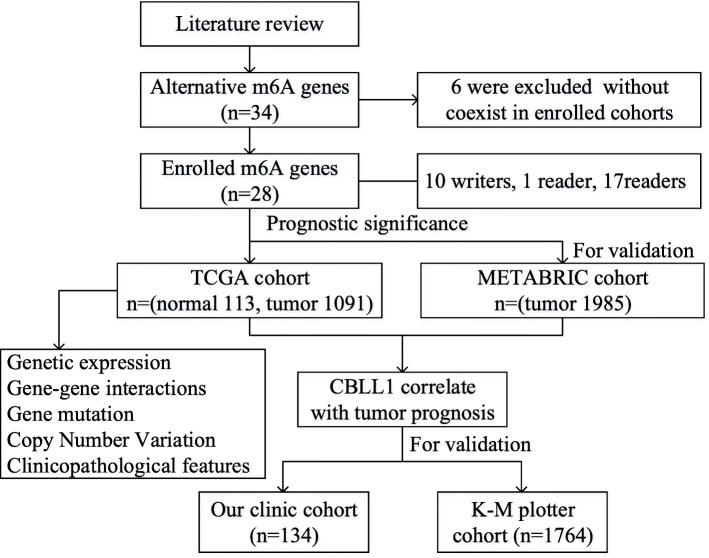
Flow chart showing the m6A modification selection and analysis process.

The TCGA data were downloaded from the TCGA breast cancer cohort within the Genomic Data Common (GDC) data portal (https://portal.gdc.cancer.gov/) The dataset contains clinical data, genetic mutation, copy number variation (CNV), and m6A regulator expression data. For validation, we obtained independent gene expression and survival data from the METABRIC (http://www.cbioportal.org/), Kaplan-Meier Plotter websites (https://kmplot.com/analysis/) and one clinic cohort. In the TCGA cohort, 1091 BC samples and 113 normal adjacent tissues were enrolled. Besides, a total of 1985 BC samples from the METABRIC cohort, 1764 BC samples from the KM plotter website cohort and 134 BC samples from one clinical cohort were enrolled and analyzed in the validation sets. (see Fig. 1) The detailed clinicopathological parameters of the BC patients are shown in Table 1.

**Table 1:** Clinicopathological features of breast cancer patients from different databases (n)

Parameters	Total	TCGA	METABRIC	Clinical cohort
Age	<=60	601	876	81
	> 60	490	1109	53
localization	Left breast	567	973	58
	Right breast	523	897	71
	others	1	115	5
T	T1	280	854	33
	T2	631	1004	84
	T3	137	94	11
	T4	40	7	2
	others	3	26	4
N	N0	535	1037	67
	N1	360	624	36
	N2	120	226	18
	N3	76	91	13
	Others	NS	7	NS
pTNM stage	I	182	552	19
	II	622	1094	75
	III	249	312	36
	Others	38	22	4
HER2 status	HER2 positive	182	247	42
	HER2 negative	753	1733	92
PAM50 molecular typing	Basal	171	329	NS
	Her2	78	240	NS
	Luminal A	497	720	NS
	Luminal B	196	490	NS
	Normal	36	200	NS
	Others	113	6	NS
All patients	total number	1091	1985	134

*NS* not shown, *TCGA* The Cancer Genome Atlas, *METABRIC* Molecular Taxonomy of Breast Cancer International Consortium. Databases including: TCGA cohort, METABRIC database, and one Clinical database. K-M plotter website database is not listed here (from the website: https://kmplot.com/analysis/)

Referring to the relevant literature on m6A methylation modification, 34 alternative m6A regulators were first included to be studied, and then 6 regulators were excluded since they did not coexist in the enrolled cohorts. (31 m6A regulators in the TCGA cohort and 28 m6A regulators in the METABRIC cohort, Fig. 1) Thus, a total of 28 m6A regulators were enrolled in this analysis, including 10 writers, 1 eraser and 17 readers, respectively [2, 17–22].

### Tissue microarray from clinic cohort

The BC tissue microarray (#HBreD140Su07, clinical cohort) was purchased from Shanghai Outdo Biotech CO., Ltd. (Shanghai, China) and immunohistochemically (IHC) stained for CBLL1. Specifically, the CBLL1 immunohistochemical expression in the cytoplasm was (−), (+), (++), and (+++).

### Statistical analysis

All these data and figures were analysed by using SPSS 24.0 (IBM, Chicago, USA), GraphPad Prism 8.0 (GraphPad Software, La Jolla, CA, USA) and R software (version 3.6.1). The associations between an m6A regulator CNV and the clinicopathological characteristics of the patients were analyzed with ANOVA test to conduct difference comparisons among three or more groups. Kaplan-Meier curves and the log-rank test were used to evaluate the prognostic value of m6A genes. All statistical results were significantly different at *P* value < 0.05.

## Results

### M6A expression in BC

In this study, three public cohort datasets and one clinical dataset were included, which were as follows: 1091 BC cases and 113 non-tumor normal cases from the TCGA date, 1985 cases from the METABRIC date, BC cases from the KM Plotter website, and 134 BC cases from one clinical dataset.

In this study, the following 28 m6A regulators were studied: 10 writers were METTL3, METTL14, METTL16, WTAP, ZC3H13, RBM15B, RBM15, CBLL1, KIAA1429, and NSUN2; 1 eraser: ALKBH5; and 17 readers: YTHDC1, YTHDC2, YTHDF1, YTHDF2, YTHDF3, FXR2, FXR1, EIF3A, EIF4G2, IGF2BP1, IGF2BP2, IGF2BP3, ELAVL1, G3BP1, HNRNPA2B1, LRPPRC, and ABCF1.

### Genetic differences between BC and adjacent normal tissues

**Fig. 2:**
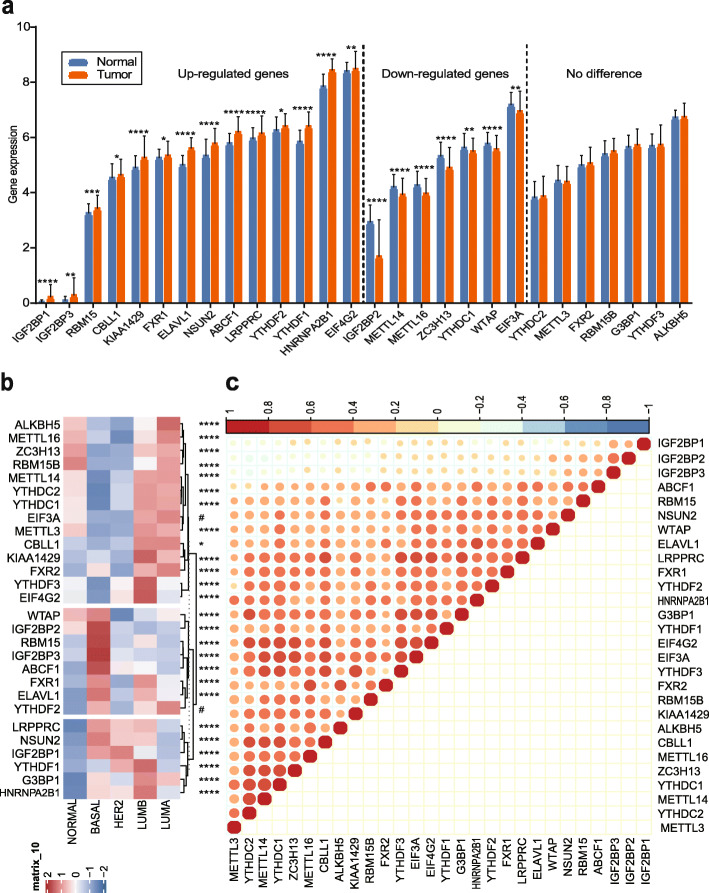
m6A regulator expression of m6A modification in breast cancer. **a** Gene expression between breast cancer tissues and adjacent normal tissues according to TCGA. **b** Genetic differences of different PAM50 molecular typing of breast cancer. **c** Correlations among the 28 enrolled m6A regulators. *P < 0.05, **P < 0.01, ***P < 0.001, ****P < 0.0001, #*P* > 0.05. TCGA: The Cancer Genome Atlas; m6A: N6-methyladenosine

Based on the TCGA data cohort, the analysis results demonstrated that 21 identified genes were differentially expressed between BC tissues and adjacent normal tissues, including 14 up-regulated genes and 7 down-regulated genes (Fig. 2a). These up-regulated genes were IGF2BP1, IGF2BP3, RBM15, CBLL1, KIAA1429, FXR1, ELAVL1, NSUN2, ABCF1, LRPPRC, YTHDF2, YTHDF1, HNRNPA2B1, and EIF4G2. These down-regulated genes were IGF2BP2, METTL14, METTL16, ZC3H13, YTHDC1, WTAP, and EIF3A. In addition, no significant differences were observed for the other seven genes (Fig. 2a).

### Genetic differences between different types of BC

Based on the TCGA cohort, the ANOVA showed that the expression of 28 m6A regulators in various BC types was significantly different (Fig. 2b). The results showed that the expression of m6A regulators was significantly correlated with different molecular typing of BC, except EIF3A and YTHDF2 (Fig. 2b, supplementary Figure 1). Specifically, IGF2BP1, IGF2BP2, and IGF2BP3 were highly expressed in TNBC, while they were expressed at low levels in HER2+, luminal A, and luminal B BC (Fig. 2b).

### Interaction of m6A regulators

By analyzing the TCGA cohort, the results indicated that all enrolled 28 studied genes exhibited gene-gene interactions. The results also showed negative gene-gene expression interaction among IGF2BP1, IGF2BP2, and IGF2BP3, and strong positive gene-gene expression interactions among the remaining 25 genes (Fig. 2c).

### Genetic mutation and copy number variations (CNVs) of the m6A regulators

**Fig. 3:**
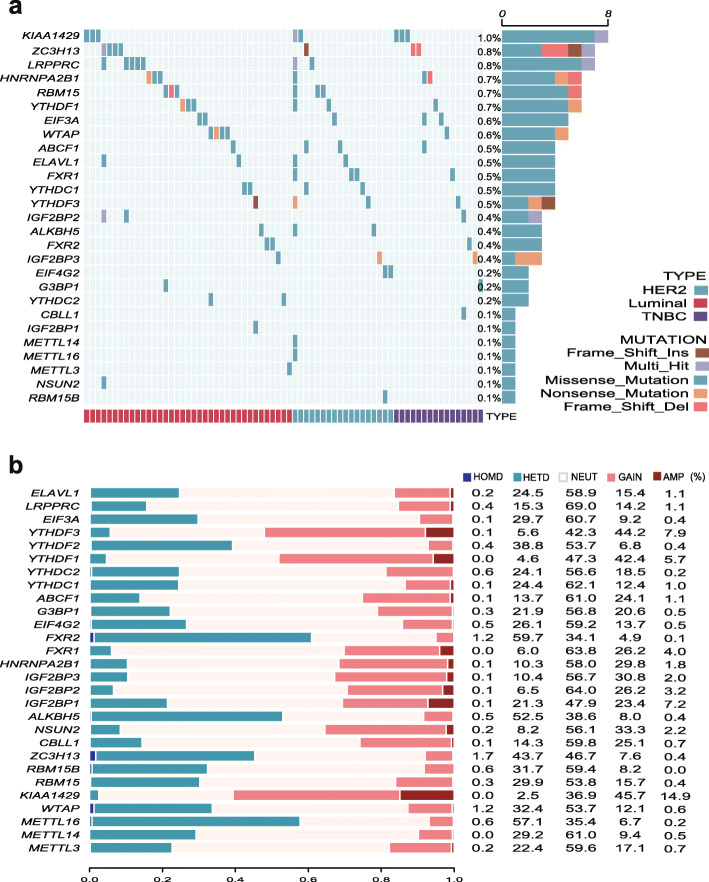
Gene mutations and CNVs of breast cancer. **a** The 28 studied genes rarely had genetic mutations, and the overall mutation frequency was only 8.45% (71/840). The mutation frequency of KIAA1429 was only approximately 1%. **b** CNV of breast cancer. AMP: amplification; HOMD: homozygous deletion; HETD: heterozygous deletion; CNV: copy number variation

According to the TCGA data cohort, the results showed that these enrolled 28 studied genes rarely had genetic mutations, and the overall mutation frequency was only 8.45% (71/840). The statistical analysis indicated that the mutation frequency of KIAA1429, which had the highest frequency, was only approximately 1%, and as for the other genes, the frequencies were below 1% (Fig. 3a). We also used COSMIC (the Catalogue Of Somatic Mutations In Cancer, https://cancer.sanger.ac.uk/cosmic/) to explore the variants of m6A regulators in BC. The results demonstrated that the mutation frequency of IGF2BP2 was 6.3%, IGF2BP3 was 6.0% and LRPPRC was 4.2%. The mutation frequencies of the remaining m6A regulators remained between 0.5% and 2.9%.

In this study, we also analyzed the following CNVs of m6a regulators: homozygous deletion (HOMD), heterozygous deletion (HETD), neural (NEUT), gain, and amplification (AMP). The top three genes with CNV gains were KIAA1429, YTHDF3, and YTHDF1; the top three genes with HOMD mutations were ZC3H13 and FXR2, and WTAP; the top three genes with HETD mutations were FXR2, METT16, and ALKBH5; and the top three genes with AMP mutations were KIAA1429, YTHDF3, and IGF2BP1 (Fig. 3b). Moreover, the results indicated a clear relationship between m6A regulators and CNVs. (Supplementary Figure 2) Supplementary Figure 2 also showes that the expression trend of some genes was from low to high. Furthermore, the expression of ABCF1, ALKBH5, ELAVL1 and FXR1 expression was gradually increased from deletion, loss, neural, gain, to amplification (Supplementary Figure 2a-d).

### Prognostic significance of m6A regulators in BC

#### Prognostic significance from TCGA data and METABRIC data

**Fig. 4:**
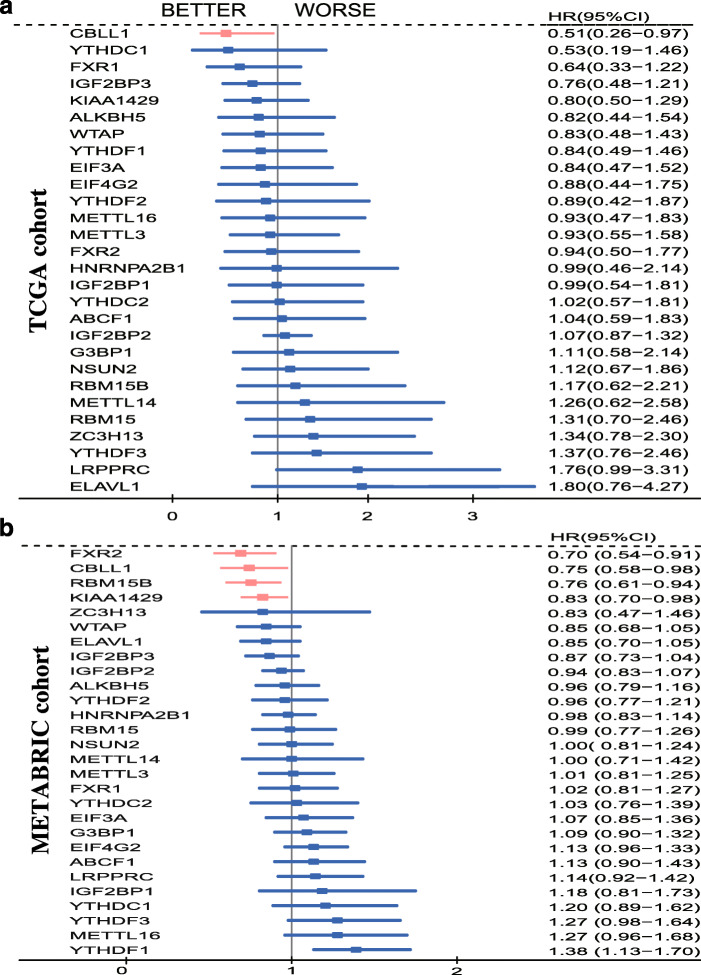
Forest plot of prognostic significance of the 28 included m6A regulators in breast cancer. **a** OS results from the TCGA cohort, **b** OS results from the METABRIC cohort. TCGA: The Cancer Genome Atlas; m6A: N6-methyladenosine; OS: overall survival

In the TCGA cohort, 816 eligible samples were selected according to the following inclusion criteria: 1. overall survival (OS) > 6 months; and 2. enrolled sample has complete stage, molecular classification and relapse-free survival (RFS) data. The Cox univariate analysis showed that BC patients with CBLL1 high expression had a better RFS than those with low CBLL1 expression. (HR95%CI = 0.51(0.26–0.97)) (Fig. 4a).

In the METABRIC cohort, the inclusion criteria of the population were as follows: 1. OS > 12 months; 2. The sample has complete grade, TNM stage and molecular classification data. The Cox univariate analysis showed that the expression of the following 4 genes was related to better OS: CBLL1 (HR95%CI = 0.70 (0.54–0.91)), FXR2 (HR95%CI = 0.75 (0.58–0.98)), RBM15B (HR95%CI = 0.76 (0.61–0.94)), and KIAA1429 (HR95%CI = 0.76 (0.61–0.94)). In contrast, YTHDF1was related to poor OS. (HR95%CI = 1.38 (1.13–1.70)) (Fig. 4b).

In summary, higher CBLL1 expression in BC was associated with better RFS in the TCGA cohort and better OS in the METABRIC cohort. That is, higher CBLL1 expression is correlated with better prognosis.

#### Relationship between CBLL1 and clinicopathological characteristics of BC

**Fig. 5:**
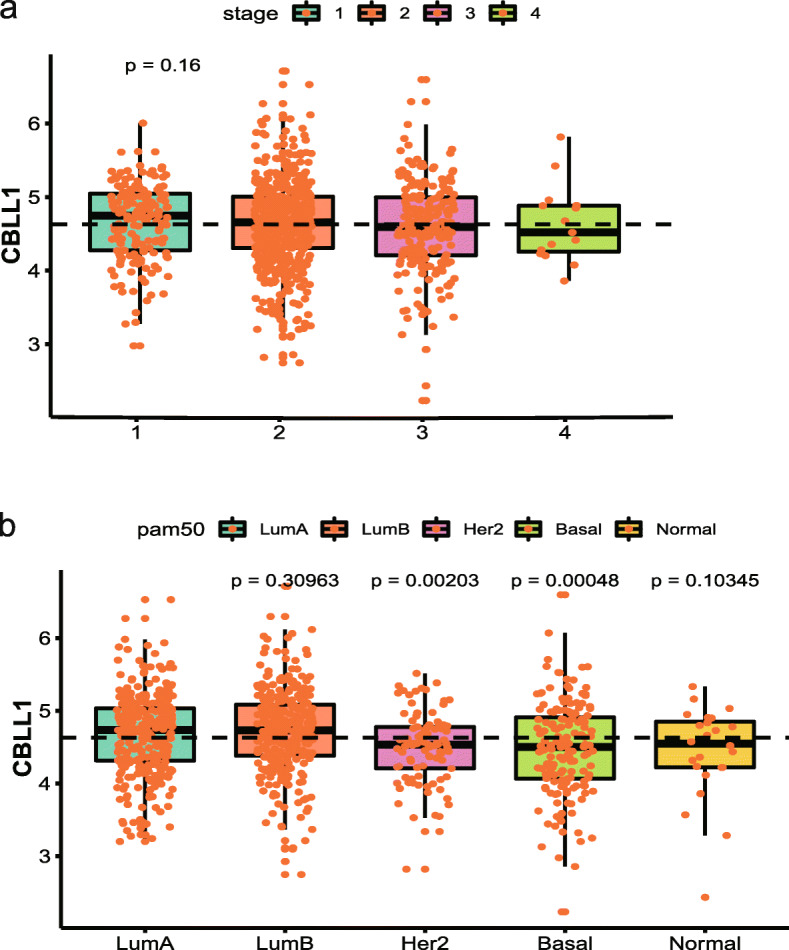
The relationship between CBLL1 and the clinicopathological characteristics of breast cancer. **a** CBLL1 with stage. **b** CBLL1 with PAM50 molecular typing

Based on the TCGA cohort, the results showed that CBLL1 expression was not significantly different in different stages of BC (*P* = 0.16, Fig. 5a). Additionally, we analysed the relationship between CBLL1 and the PAM50 molecular subtypes of BC. The expression of CBLL1 in luminal BC was significantly higher than that in HER2 positive BC and TNBC (*P* < 0.05, Fig. 5b). However, CBLL1 expression in luminal A BC was not significantly different from that in luminal B and PAM50-normal BC. (*P* > 0.05, Fig. 5b).

#### Prognostic significance of CBLL1 in BC

**Fig. 6:**
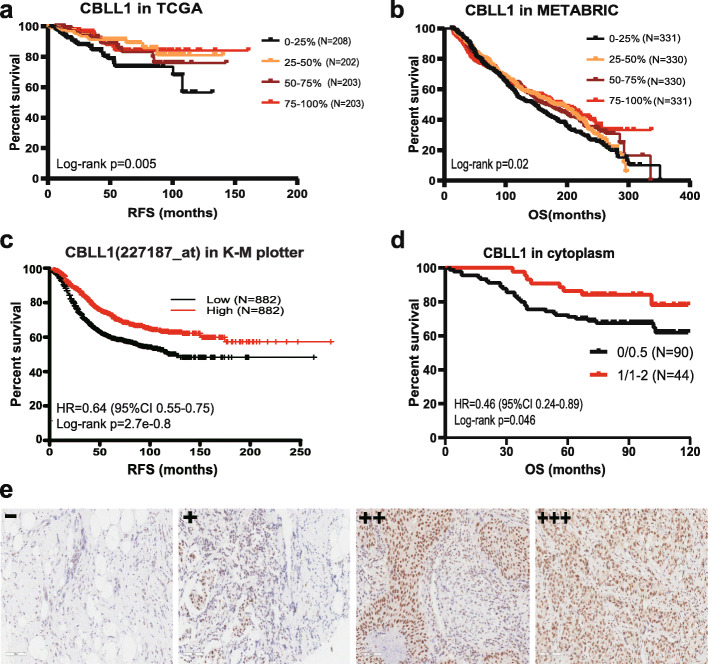
Prognostic significance of CBLL1 in breast cancer. **a** Association of CBLL1 RNA expression with RFS in the TCGA cohort. **b** Association of CBLL1 RNA expression with OS in the METABRIC cohort. **c** Association of CBLL1 RNA expression with RFS in the K-M plotter cohort. **d** Association of CBLL1 (immunohistochemistry staining) with OS in the clinical dataset. **e** CBLL1Immunohistochemistry, including (−), (+), (++), (+++); (−)/(+): low expression; (++)/(+++): high expression. OS: overall survival; RFS: relapse-free survival; TCGA: The Cancer Genome Atlas

From the TCGA cohort, a total of 816 samples were included to analyse the relationship between CBLL1 expression and the prognosis of BC patients. The results showed that patients with high CBLL1 expression had better RFS (*P* = 0.005, Fig. 6a). In the METABRIC cohort, the results of the analysis with the quartile method showed that patients with high CBLL1 expression had better OS with the quartile method involved (*P* = 0.02, Fig. 6b). Moreover, based on that cohort in the Kaplan-Meier Plotter website, we confirmed that high CBLL1(affymetrix ID: 227187_at) expression is correlated with better RFS (HR = 0.64 (0.55–0.75), log-rank *p* = 2.7e− 08) (See Fig. 6c). In addition, the analysis of 134 selected clinical samples showed that patients with high CBLL1 expression in the cytoplasm also had better OS (HR = 0.46 (95%CI 0.24–0.89), log-rank *p* = 0.046) (See Fig. 6d). In this study, CBLL1 −/+ was defined as low expression, and CBLL1++/+++ was defined as high expression (See Fig. 6e).

#### Functional analysis of CBLL1 in luminal BC

In a previous study, Hakai, as a coregulator of oestrogen receptor alpha, was found to play a negative role in the development and progression of BC cells [23]. Thus, we performed functional analysis of CBLL1 in ER- or PR-positive BC. Based on the TCGA cohort, a total of 597 ER- or PR-positive and HER2-negative BC patients patients were included according to the inclusion criteria. Altogether, 521 genes with differential gene expression (log FC > 0.8 or < − 0.3) were included in the analysis.

**Fig. 7:**
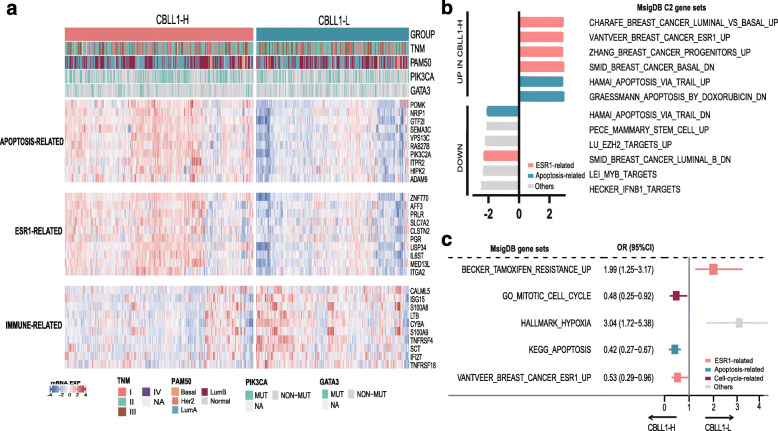
Functional Analysis of CBLL1. **a** Relationship between CBLL1 and signalling pathways. **b** GSEA of CBLL1. **c** ssGSEA of CBLL1. GSEA: gene set enrichment analysis; ssGSEA: single-sample gene set enrichment analysis

A total of 597 patients was were divided into the CBLL1-high (CBLL1-H) and CBLL1-low (CBLL1-L) groups according to the median value of CBLL1 expression. The heat map compared the pathological features and signalling pathway features of BC in the CBLL1 high-expression group with those in the CBLL1-low expression group. The following parameters were observed in these two groups: pTNM staging, PAM50, PIK3CA, GATA3, apoptosis-related pathways, ESR1-related pathways, and immune-related pathways (Fig. 7a). The expression of the following apoptosis regulators was significantly different in the CBLL1-H and CBLL1-L group, as follows: POMK, NRIP1, GTF2I, SEMA3C, VPS13C, RAB27B, PIK3C2A, ITPR2, HIPK2, and ADAM9. In addition, the expression of ESR1 regulators,including ZNF770, AFF3, PRLR, SLC7A2, and CLSTN2, was also significantly different in CBLL1-H and CBLL1-L group. Furthermore, immune regulators, including CALML5, ISG15, S100A8, LTB, CYBA, S100A9, TNFRSF4, SCT, and IFI27, were significantly different between the CBLL1-H and CBLL1-L groups (Fig. 7a).

Gene set enrichment analysis (GSEA) demonstrated that the CBLL1-related differentially expressed genes were significantly enriched in the ESR1-related signalling pathway, cell apoptosis related pathway and immune-related pathway. In particular, CBLL1 expression promoted the upregulation of genes related to ESR1 and cell apoptosis-related pathways (Fig. 7b). Moreover, single-sample gene set enrichment analysis (ssGSEA) demonstrated that low CBLL1 expression increased the occurrence of tamoxifen resistance and tumor-associated hypoxia. High CBLL1 expression induced apoptosis, mitotic cell death and the upregulation of ESR1 (Fig. 7c).

## Discussion

Based on the TCGA cohort, the METABRIC cohort, the K-M plotter website cohort and one clinical cohort, this article first analysed the expression of m6A regulators in BC and the effects of m6A modification on the biological behaviour of BC. Research has shown that m6A RNA modification regulates certain signalling pathways via methylation transferases, demethylation enzymes, and reader effectors and thus affects the differential expression of genes between human BC and normal tissue, as well as the molecular typing, genetic mutations, and genome CNVs of BC. In addition, m6A regulator expression was correlated with the clinicopathological characteristics, tumor drug sensitivity, RFS and OS of BC. Importantly, CBLL1 was a protective factor of prognosis in BC.

This study demonstrated that the expression of some m6A regulators expressions was significantly different between BC tissues and adjacent normal tissues. Previous studies have shown that m6A modification plays an important role in a variety of tumors, such as gastric cancer, glioblastoma, kidney cancer, and BC [23–26]. In this study, 21 m6A regulators were differentially expressed, including 14 upregulated genes and 7 downregulated genes. The expression of CBLL1 expression was upregulated in lung cancer and BC compared with adjacent tissues [27]. Consistent with the findings of the above study, our research also confirmed that CBLL1 was up-regulated in BC. KIAA1429 expression was upregulated in BC tissue and regulated tumor proliferation and tumor differentiation [28]. This stydy also confirmed that KIAA1429 was upregulated in BC. Previous studies showed that METTL3 was downregulated in BC cell lines and tissues [7]. However, METTL3 expression was no differentially expressed between cancer tissues and normal tissues. ALKBH5 and G3BP1 have been found to be overexpressed in BC; however, they did not show differential expression in our study [12, 29]. To date, the expression of m6A regulator is controversial. The possible reasons are as follows:1. fewer samples were included in previous studies; 2. most previous samples were cell lines; and 3. no specific molecular typing classification was used.

In addition, m6A RNA regulators exhibit gene-gene interactions and have different gene expression patterns in different molecular types of BC. Previous studies have shown that crosslinks among different m6A regulators, affect tumor pathogenesis and differentiation [2]. We also observed that m6A regulators, except EIF3A and YTHDF2, were clearly correlated with different molecular types of BC. Of note, IGF2BP1, IGF2BP2, and IGF2BP3 expressions were upregulated in TNBC and downregulated in HER2+, luminal A, and luminal B BC. CBLL1 expression was upregulated in ER-positive breast cancer [23]. In other words, the above relevant regulators may participate in the regulation of molecular typing.

Futhermore, our research indicated that the mutation frequencies of m6A regulators were low in BC, but the CNV mutation frequencies of m6A regulators were high and related to gene expression. Rui et al. confirmed that the mutation frequencies of all m6A regulators were lower than 1.1% in glioma [25]. The results of one pan-cancer study also confirmed that the mutation frequencies of m6A regulators were very low in tumors, ranging from 0.02 to 8.07% [30]. Similarly, the mutation frequencies of the 28 included genes were less than 1%, and that of the top gene KIAA1429 was only 1.0%. Based on the data presented in this research, gene mutation have little impacts on m6A modifications. However, another study of renal cancer confirmed that the mutation frequency of the m6A regulator YTHDC2 was 55.11% and that of METTL3 was 30.11% [24]. That is, gene mutations were different in various cancers. In gastric cancer, m6A modifications are mediated by the CNV deletions of ELAVL1, YTHDF2 and FMR1, which then affects tumor formation [26]. As described above, the m6A regulators of BC also had a high frequency of CNVs. For example, KIAA1429, YTHDF3, and YTHDF1 had higher CNV gain frequencies of over 40%. The CNV HETD frequencies of FXR2, METT16, and ALKBH5 were all higher than 50%. In addition, the expression of m6A regulators correlated with CNVs. In particular, the expression of ABCF1, ALKBH5, ELAVL1 and FXR1 increased with increasing CNVs. Therefore, based on former conclusions, we can speculate that genomic CNVs play a stronger role in m6A modification compared with gene mutations.

Meanwhile, this result indicated that the m6A regulator CBLL1 had no correlation with TNM stage in BC. Similarly, CBLL1 had no correlation with TNM stage in lung cancer [27]. This result may indicate that CBLL1 does not affect tumor stage. However, WTAP, RBM15, YTHDF, and ALBKH5 had strong correlations with tumor stage and 1p/19q codeletion in glioma [25]. This m6A regulator was correlated with the nuclear grade of kidney cancer [24]. Of course, further experiments are needed to verify whether CBLL1 can regulate the malignant phenotype of tumor cells.

This study found that m6A regulator is related to prognosis in BC. CBLL1 expression correlated with poor prognosis in lung cancer [27]. However, CBLL1 was correlated with good prognosis in BC, according to the TCGA, METABRIC, K-M plotter and one clinical cohorts. Similarly, previous studies have found that CBLL1, as an E3 ubiquitin ligase, inhibits ER pathway activity by binding to an ER coactivator and then further inhibits the proliferation and differentiation of BC cells [23]. The potential reason may be that CBLL1 inhibited tumor metastasis, enhanced cell apoptosis or increased drug sensitivity. It is also important to note that CBLL1 has low tissue specificity, according to data from the Human Protein Atlas (https://www.proteinatlas.org/ENSG00000105879-CBLL1). Certainly, further work is needed to enhance the accuracy of this inference. Nevertheless, high CBLL1 expression is a protective factor for BC patients. In addition, some m6A regulators were associated with prognosis. For example, IGF2BP1 expression implies a poor prognosis in ovarian, liver and lung cancers [31–33]. METTL3 expression correlated with brain metastasis and worse prognosis in lung cancer [34].

Furthermore, functional analysis indicated that CBLL1 may affect the occurrence, development and drug resistance of BC by regulating various pathways. CBLL1 expression was associated with apoptosis-related pathways, ESR1-related pathways, and immune-related pathways. That is, CBLL1 plays a vital role in regulating these signalling pathways or their related genes, such as mediating POMK and NRIP1 of the apoptosis-related pathway, ZNF770 and AFF3 of the ESR1-related pathway, and CALML5 and ISG15 of the immune-related pathway. In the CBLL1 high expression group, the ESR1-related pathway was upregulated. These results are not consistent with the results of previous studies showing that CBLL1 inhibits ER pathway activity by binding to ER coactivators [23], probably because of these genes are regulated by different cancer pathways. In addition, the apoptotic pathway was also upregulated in the CBLL high expression group and may regulate the cell cycle and apoptosis of BC. Nevertheless, in addition to CBLL1, which indicates a better prognosis, we suspect that the activation of the above pathways might be the reason for the prolonged survival time of patients. Furthermore, our study demonstrated that low CBLL1 expression was related to the tamoxifen resistance pathway; that is, resistance to tamoxifen was more likely to occur in CBLL1-low patients. Therefore, other nonsteroidal endocrine therapies should be considered in CBLL1-low expression patients. Therefore, CBLL1 could be a suitable and attractive target for new cancer therapies.

Of course, the study also has some limitations, as follows: Only 28 m6A regulators were analysed in the current cohorts. However, in this study, multiple cohorts were firstly used to study the correlation between m6A modification and BC. The genes involved in thisstudy are not comprehensive; for example, one eraser of m6A, FTO, was excluded due to a lack of detection in the enrolled cohorts. This research focused on survival, and more studies are needed to further explore the specific mechanisms and detailed pathways.

## Conclusions

In conclusion, this study showed that m6A modifications affect the tumorigenesis, molecular typing, genetic mutations, CNVs, and prognosis of BC patients. Higher CBLL1 expression was correlated with better prognosis in BC. CBLL1 can be considered a novel and excellent target for antitumor therapy.

## Supplementary Information

**Additional file 1: Supplementary figure 1.** The relationship between 28 m6A regulators and different PAM50 molecular typing of breast cancer. PAM50 molecular typing including normal, basal, luminal A, luminal B, HER2. M6A: N6-methyladenosine.

**Additional file 2: Supplementary figure 2.** The relationship between 28 m6A regulators and CNV of breast cancer. CNV: Copy Number Variation. (a-q) we listed 16 genes of 28 enrolled m6A regulators. M6A: N6-methyladenosine.

## Data Availability

Three public datasets were obtained from TCGA (https://portal.gdc.cancer.gov/), METABRIC (http://www.cbioportal.org/), and Kaplan-Meier Plotter websites (https://kmplot.com/analysis/). The clinic cohort data was analyzed by Shanghai Outdo Biotech CO., Ltd. (Shanghai, China).

## Abbreviations

BC: Breast carcinoma or breast cancer
M6A: N6-methyladenosine
TCGA: The Cancer Genome Atlas
METABRIC: Molecular Taxonomy of Breast Cancer International Consortium
CNV: Copy number variation
UTR: Untranslated region
NEUT: Neural
HOMD: Homozygous deletion
HETD: Heterozygous deletion
ssGSEA: Single-sample gene set enrichment analysis
GSEA: Gene set enrichment analysis
DFS: Disease-free survival
RFS: Relapse-free survival
OS: Overall survival

## References

[1] Bray F, Ferlay J, Soerjomataram I, Siegel RL, Torre LA, Jemal A (2018). Global cancer statistics 2018: GLOBOCAN estimates of incidence and mortality worldwide for 36 cancers in 185 countries. CA Cancer J Clin.

[2] He L, Li H, Wu A, Peng Y, Shu G, Yin G (2019). Functions of N6-methyladenosine and its role in cancer. Mol Cancer.

[3] Chen B, Li Y, Song R, Xue C, Xu F (2019). Functions of RNA N6-methyladenosine modification in cancer progression. Mol Biol Rep.

[4] Kim HY, Ha Thi HT, Hong S (2018). IMP2 and IMP3 cooperate to promote the metastasis of triple-negative breast cancer through destabilization of progesterone receptor. Cancer Lett.

[5] McMullen ER, Gonzalez ME, Skala SL, Tran M, Thomas D, Djomehri SI (2018). CCN6 regulates IGF2BP2 and HMGA2 signaling in metaplastic carcinomas of the breast. Breast Cancer Res Treat.

[6] Toyama T, Kondo N, Endo Y, Sugiura H, Yoshimoto N, Iwasa M, Takahashi S, Fujii Y, Yamashita H (2012). High expression of microRNA-210 is an independent factor indicating a poor prognosis in Japanese triple-negative breast cancer patients. Jpn J Clin Oncol.

[7] Wang H, Xu B, Shi J (2020). N6-methyladenosine METTL3 promotes the breast cancer progression via targeting Bcl-2. Gene.

[8] Wu L, Wu D, Ning J, Liu W, Zhang D (2019). Changes of N6-methyladenosine modulators promote breast cancer progression. BMC Cancer.

[9] Yamaga R, Ikeda K, Horie-Inoue K, Ouchi Y, Suzuki Y, Inoue S (2013). RNA sequencing of MCF-7 breast cancer cells identifies novel estrogen-responsive genes with functional estrogen receptor-binding sites in the vicinity of their transcription start sites. Horm Cancer.

[10] Yi D, Wang R, Shi X, Xu L, Yilihamu Y, Sang J (2020). METTL14 promotes the migration and invasion of breast cancer cells by modulating N6methyladenosine and hsamiR146a5p expression. Oncol Rep.

[11] Zang XP, Pento JT, Tari AM (2008). Wilms’ tumor 1 protein and focal adhesion kinase mediate keratinocyte growth factor signaling in breast Cancer cells. Anticancer Res.

[12] Zhang C, Zhi WI, Lu H, Samanta D, Chen I, Gabrielson E, Semenza GL (2016). Hypoxia-inducible factors regulate pluripotency factor expression by ZNF217- and ALKBH5-mediated modulation of RNA methylation in breast cancer cells. Oncotarget.

[13] Huang H, Weng H, Chen J (2020). M (6) a modification in coding and non-coding RNAs: roles and therapeutic implications in Cancer. Cancer Cell.

[14] Shulman Z, Stern-Ginossar N (2020). The RNA modification N (6)-methyladenosine as a novel regulator of the immune system. Nat Immunol.

[15] Ohashi R, Sangen M, Namimatsu S, Takei H, Naito Z (2017). IMP3 contributes to poor prognosis of patients with metaplastic breast carcinoma: a clinicopathological study. Ann Diagn Pathol.

[16] Sjekloca N, Tomic S, Mrklic I, Vukmirovic F, Vuckovic L, Lovasic IB (2020). Prognostic value of IMP3 immunohistochemical expression in triple negative breast cancer. Medicine (Baltimore).

[17] Chen XY, Zhang J, Zhu JS (2019). The role of m (6) a RNA methylation in human cancer. Mol Cancer.

[18] Dai D, Wang H, Zhu L, Jin H, Wang X (2018). N6-methyladenosine links RNA metabolism to cancer progression. Cell Death Dis.

[19] Edupuganti RR, Geiger S, Lindeboom RGH, Shi H, Hsu PJ, Lu Z, Wang SY, Baltissen MPA, Jansen PWTC, Rossa M, Müller M, Stunnenberg HG, He C, Carell T, Vermeulen M (2017). N (6)-methyladenosine (m (6) a) recruits and repels proteins to regulate mRNA homeostasis. Nat Struct Mol Biol.

[20] He L, Li J, Wang X, Ying Y, Xie H, Yan H, Zheng X, Xie L (2018). The dual role of N6-methyladenosine modification of RNAs is involved in human cancers. J Cell Mol Med.

[21] Ianniello Z, Fatica A (2018). N6-Methyladenosine Role in Acute Myeloid Leukaemia. Int J Mol Sci.

[22] Sun T, Wu R, Ming L (2019). The role of m6A RNA methylation in cancer. Biomed Pharmacother.

[23] Makdissi FB, Machado LV, Oliveira AG, Benvenuti TT, Katayama ML (2009). Expression of E-cadherin, snail and Hakai in epithelial cells isolated from the primary tumor and from peritumoral tissue of invasive ductal breast carcinomas. Braz J Med Biol Res.

[24] Zhou J, Wang J, Hong B, Ma K, Xie H, Li L, Zhang K, Zhou B, Cai L, Gong K (2019). Gene signatures and prognostic values of m6A regulators in clear cell renal cell carcinoma – a retrospective study using TCGA database. Aging.

[25] Chai R-C, Wu F, Wang Q-X, Zhang S, Zhang K-N, Liu Y-Q, Zhao Z, Jiang T, Wang YZ, Kang CS (2019). m6A RNA methylation regulators contribute to malignant progression and have clinical prognostic impact in gliomas. Aging.

[26] Zhang B, Wu Q, Li B, Wang D, Wang L, Zhou YL (2020). m (6) A regulator-mediated methylation modification patterns and tumor microenvironment infiltration characterization in gastric cancer. Mol Cancer.

[27] Hui L, Zhang S, Wudu M, Ren H, Xu Y, Zhang Q, Qiu X (2019). CBLL1 is highly expressed in non-small cell lung cancer and promotes cell proliferation and invasion. Thorac Cancer.

[28] Qian JY, Gao J, Sun X, Cao MD, Shi L, Xia TS, Zhou WB, Wang S, Ding Q, Wei JF (2019). KIAA1429 acts as an oncogenic factor in breast cancer by regulating CDK1 in an N6-methyladenosine-independent manner. Oncogene.

[29] Zhang CH, Wang JX, Cai ML, Shao R, Liu H, Zhao WL (2019). The roles and mechanisms of G3BP1 in tumour promotion. J Drug Target.

[30] Li Y, Xiao J, Bai J, Tian Y, Qu Y, Chen X, Wang Q, Li X, Zhang Y, Xu J (2019). Molecular characterization and clinical relevance of m (6) a regulators across 33 cancer types. Mol Cancer.

[31] Gong F, Ren P, Zhang Y, Jiang J, Zhang H (2016). MicroRNAs-491-5p suppresses cell proliferation and invasion by inhibiting IGF2BP1 in non-small cell lung cancer. Am J Transl Res.

[32] Muller S, Bley N, Glass M, Busch B, Rousseau V, Misiak D (2018). IGF2BP1 enhances an aggressive tumor cell phenotype by impairing miRNA-directed downregulation of oncogenic factors. Nucleic Acids Res.

[33] Hammerle M, Gutschner T, Uckelmann H, Ozgur S, Fiskin E, Gross M (2013). Posttranscriptional destabilization of the liver-specific long noncoding RNA HULC by the IGF2 mRNA-binding protein 1 (IGF2BP1). Hepatology.

[34] Wang H, Deng Q, Lv Z, Ling Y, Hou X, Chen Z, Dinglin X, Ma S, Li D, Wu Y, Peng Y, Huang H, Chen L (2019). N6-methyladenosine induced miR-143-3p promotes the brain metastasis of lung cancer via regulation of VASH1. Mol Cancer.

