# Supplementary figures and images for: Expression and clinical prognostic value of m6A RNA methylation modification in breast cancer
Source: Biomark Res. 2021 Apr 29;9:28. doi: 10.1186/s40364-021-00285-w (PMC8082898; doi:10.1186/s40364-021-00285-w)

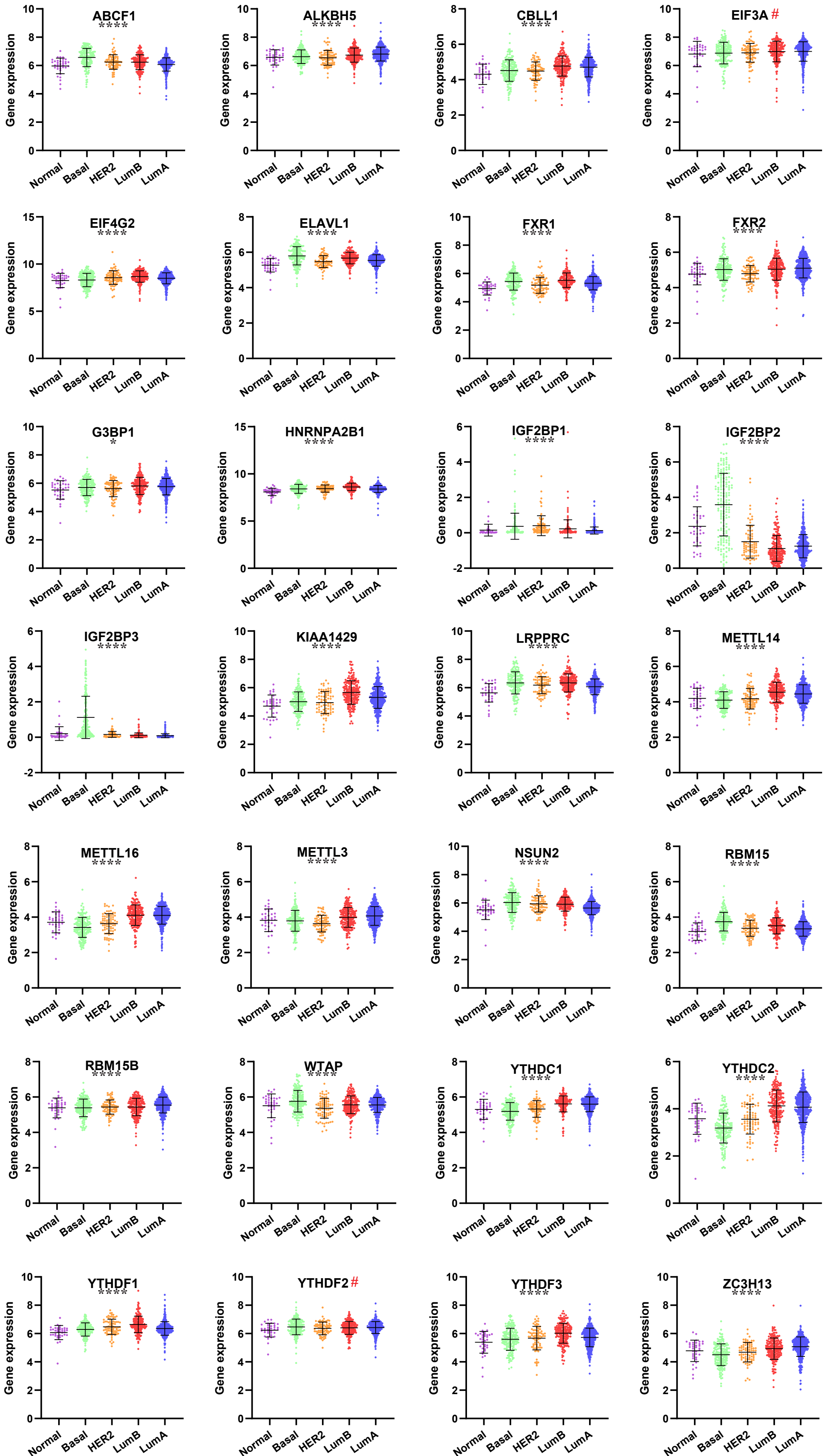

Supplement: Supplementary file 1 — Additional file 1: Supplementary figure 1. The relationship between 28 m6A regulators and different PAM50 molecular typing of breast cancer. PAM50 molecular typing including normal, basal, luminal A, luminal B, HER2. M6A: N6-methyladenosine. [file 40364_2021_285_MOESM1_ESM.pdf]

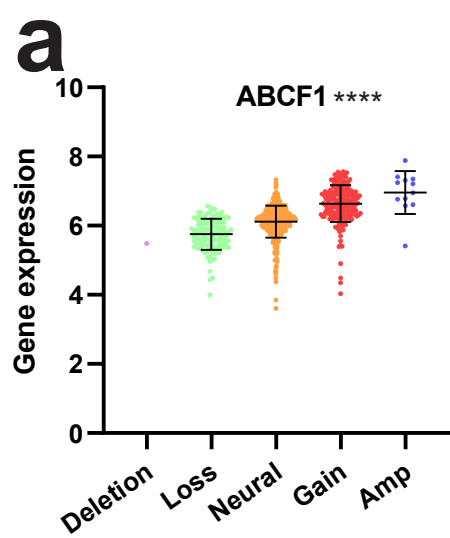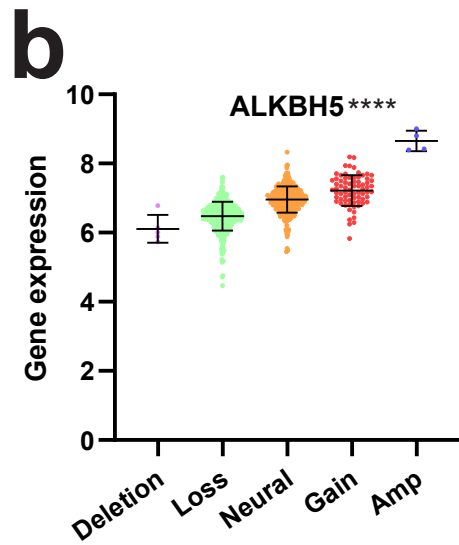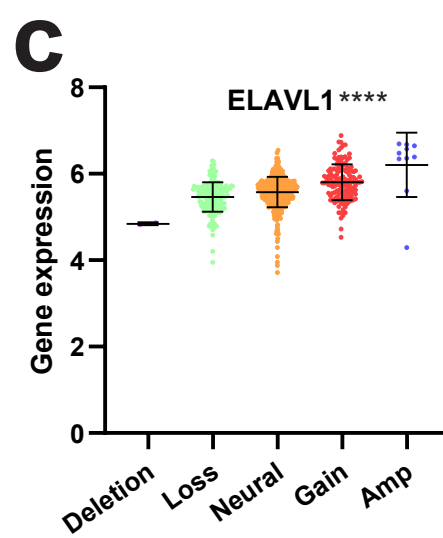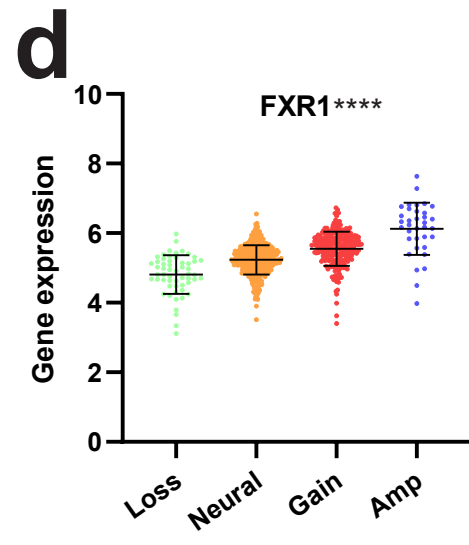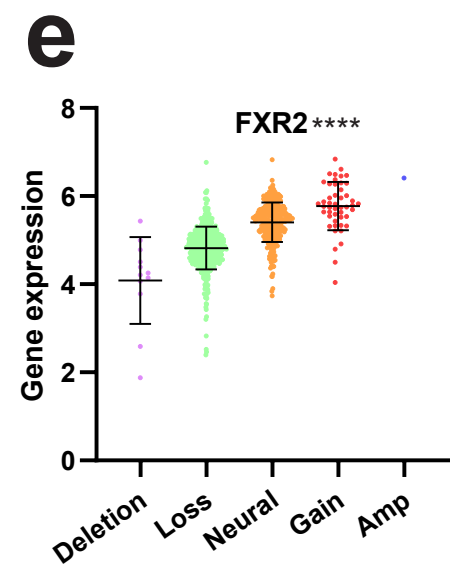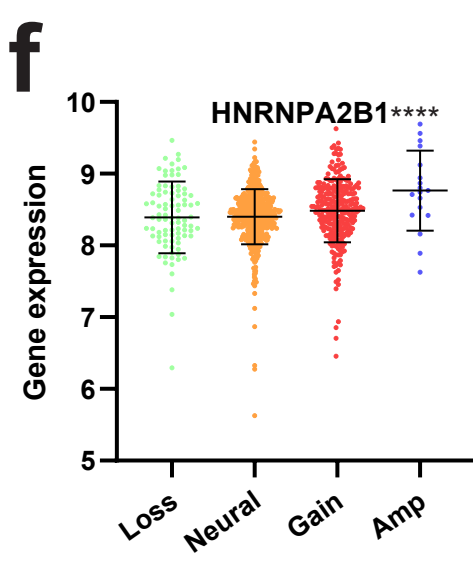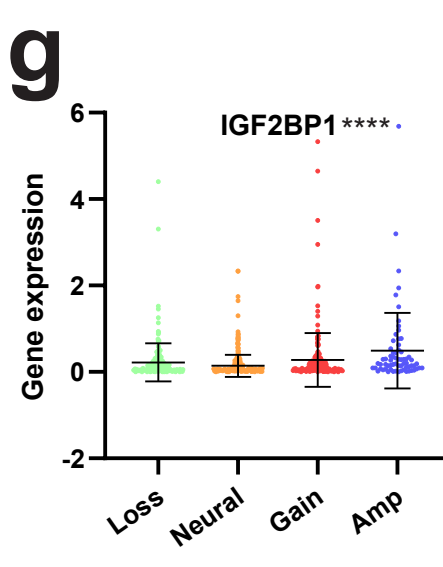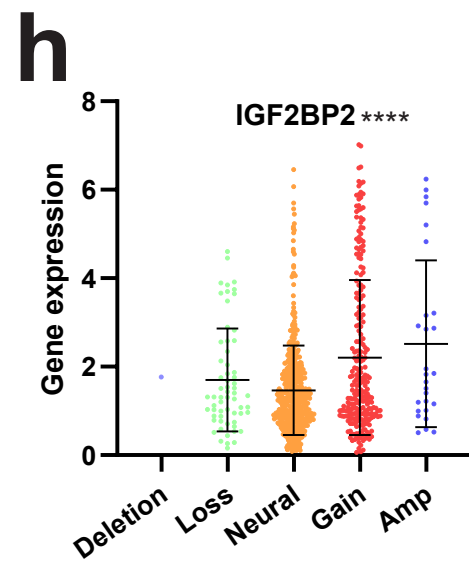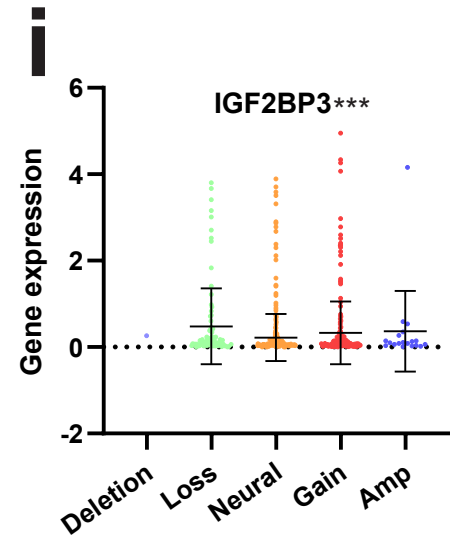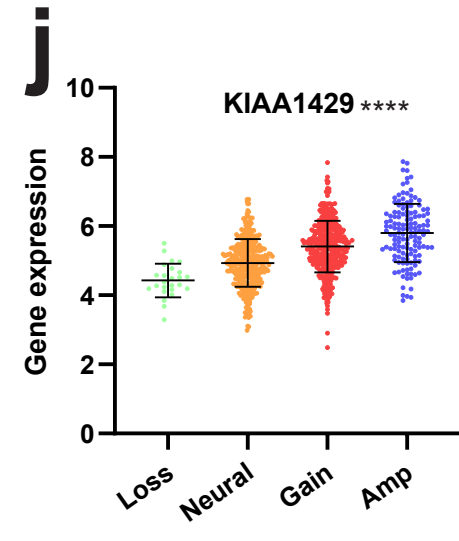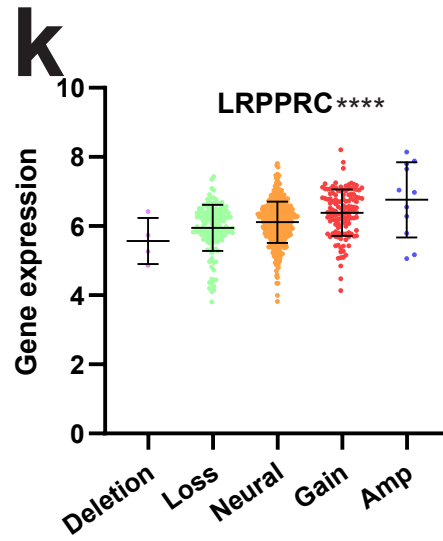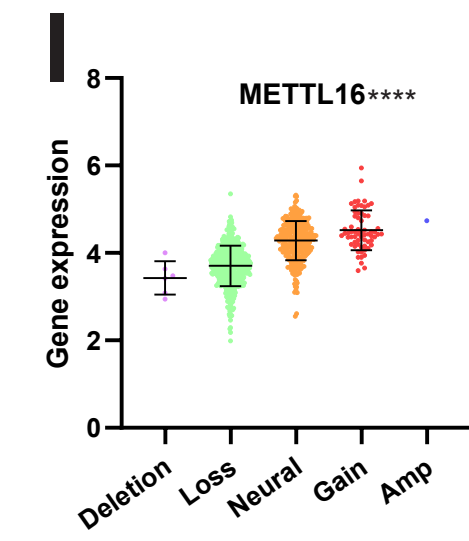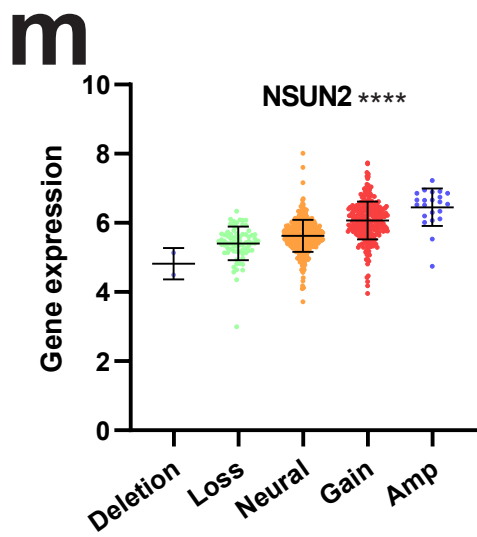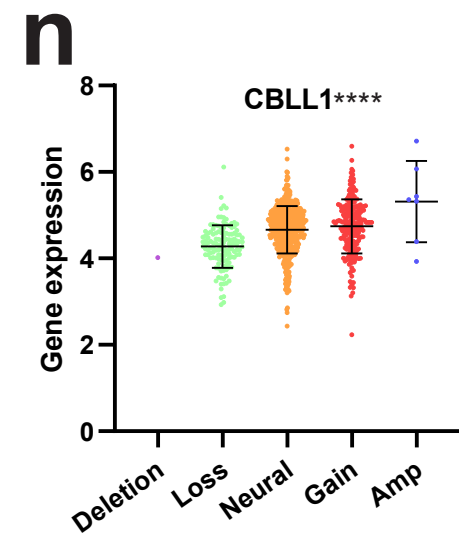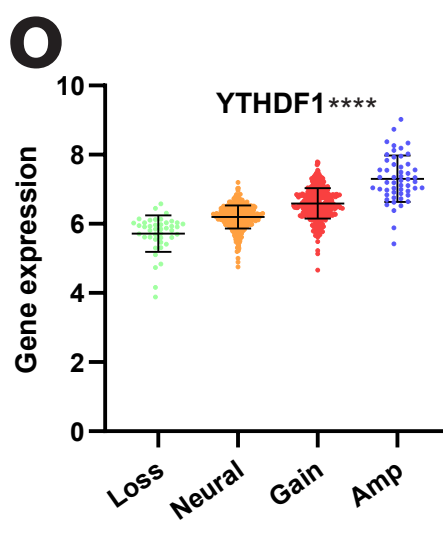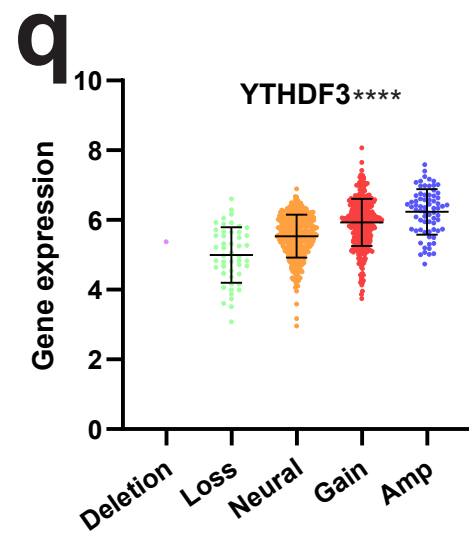

Supplement: Supplementary file 2 — Additional file 2: Supplementary figure 2. The relationship between 28 m6A regulators and CNV of breast cancer. CNV: Copy Number Variation. (a-q) we listed 16 genes of 28 enrolled m6A regulators. M6A: N6-methyladenosine. [file 40364_2021_285_MOESM2_ESM.pdf]
